# Genomic prediction for grain yield and biotic stress resistance in field pea (*Pisum sativum* L.)

**DOI:** 10.3389/fpls.2026.1739804

**Published:** 2026-04-01

**Authors:** Adnan Riaz, Yongjun Li, Babu Ram Pandey, Alem Gebremedhin, Shiva Azizinia, Shimna Sudheesh, Zibei Lin, Joshua Fanning, Garry Rosewarne, Matthew J. Hayden, Sukhjiwan Kaur

**Affiliations:** 1 Agriculture Victoria, Department of Energy, Environment and Climate Action, Bundoora, VIC, Australia; 2 Agriculture Victoria, Grains Innovation Park, Horsham, VIC, Australia; 3 School of Applied Systems Biology, La Trobe University, Bundoora, VIC, Australia; 4 Sugar Research Australia, Brisbane, QLD, Australia

**Keywords:** biotic stress, field pea, genomic selection, genotype × environment interaction, yield

## Abstract

Field pea (*Pisum sativum* L.) is a nutritionally important pulse crop that contributes to food security, sustainable cropping systems, and the growing demand for plant-based proteins. However, genetic gain for complex traits such as grain yield and disease resistance remains limited under conventional breeding, particularly in the face of climate change and evolving biotic stresses. Genomic selection (GS) is a promising approach to accelerate genetic improvement, yet its large-scale evaluation in large-scale breeding programs has been limited. Here, we present the first comprehensive assessment of GS in the Australian National Field Pea Breeding Program, using a decade (2013–2022) of multi-environment data from 3,199 advanced lines and cultivars. Six key traits were analyzed, including grain yield (GY) and resistance to major diseases, including ascochyta blight, bacterial blight, downy mildew, pea seed-borne mosaic virus (PSbMV), and bean leaf roll virus (BLRV). Lines were genotyped using a multispecies Pulse 30K SNP array. Genomic prediction was evaluated using GBLUP models fitted with and without genotype × environment (G × E) interactions, as well as bivariate models exploiting genetic correlations between traits. Across traits and models, prediction accuracy ranged from 0.21 to 0.72. Including G × E interactions increased GY prediction accuracy by 3.03%, while bivariate models provided moderate additional gains by leveraging correlations with disease resistance traits. Overall, our results demonstrate that GS can be integrated effectively into a field pea breeding program to enhance disease resistance and stabilize yields across environments.

## 1 Introduction

The growing global demand for plant-based proteins, combined with the increasing impacts of climate change, presents challenges for sustainable food production systems (Tilman et al., 2011; Van Dijk et al., 2021). Field pea (*Pisum sativum* L.), often called the “poor man’s meat”, is an affordable, nutrient-rich crop with high protein content (16–32%), starch (30–50%), fiber (6–13%), vitamins, minerals, and prebiotic carbohydrates, with low fat (~1%) (Amarakoon et al., 2012; Arnoldi et al., 2015). Additionally, when incorporated in the farming system, this self-pollinating cool-season legume helps reduce disease pressure, disrupts pest and weed cycles, and promotes biological nitrogen fixation, thereby reducing dependence on synthetic fertilizers and pesticides (Lake et al., 2021; Warkentin et al., 2024). Furthermore, field pea helps prevent erosion, conserves moisture, and improves harvestability, making it a sustainable and preferred pulse choice for farmers. However, climate change poses a threat to pea production, causing shifts in sowing times that, in turn, increase the risk of disease outbreaks (Foyer et al., 2016). Field pea is the most affected crop by climate change in Australia, with yield reductions ranging from 12% to 45% under various climate scenarios and locations (Anwar et al., 2015). Hence, it is essential to develop high-yield, nutrient-dense, and climate-resilient field pea varieties to ensure sustainable yield and meet the growing demand for plant-based proteins.

In Australia, field peas were cultivated over approximately 192,000 hectares during the 2021–2022 period, producing around 261,000 tons (excluding animal feed production), with nearly 55% exported (ABARES, 2025). Field pea is widely grown in South Australia (80,000 ha) and Victoria (40,000 ha), where favorable Mediterranean-temperate climates promote optimal yields. Recently, cultivation has extended to Western Australia (35,000 ha) and New South Wales (40,000 ha), as part of efforts to adapt the crop to more marginal environments, known as expansion zones (ABARES, 2025). Despite its agronomic and economic significance, field pea cultivation has declined since 1988, and the rate of yield improvement has lagged for several reasons, including the cultivation of field pea on marginal cropping land and inadequate research investments (Lake et al., 2021). While field pea in Australia has the potential to yield up to 7 tonnes per hectare (t ha^-1^), the national average yields remain relatively low at approximately 1.4 t ha^-1^ (Lake et al., 2021; ABARES, 2025). There are significant regional differences in yields reported for 2021–2022, with Victoria and Western Australia reporting higher outputs of 1.8 and 1.7 t ha^-^¹, respectively. In contrast, New South Wales and South Australia exhibit lower yields at 1.22 and 1.06 t ha^-^¹ (ABARES, 2025). These differences highlight the impact of environmental factors, management practices, and genotype-by-environment interactions.

Achieving the full yield potential of field peas remains a major challenge due to their vulnerability to drought and diseases, which consistently threaten crops (Rubiales et al., 2015). In Australia, diseases cause an estimated annual loss of AU$23.7 million, or approximately AU$78.35 per hectare for field pea production (Murray and Brennan, 2012). The diseases include bacterial blight (caused by *Pseudomonas syringae*), downy mildew (caused by *Peronospora viciae*), and the complex of ascochyta blight (sy. Blackspot), which are especially problematic, damaging crop health and yield stability (Bretag et al., 2006; Hollaway et al., 2007; Tran et al., 2014). These diseases can result in substantial and recurring yield losses: ascochyta blight can reduce yield by up to 60%, annually causing AU$20 million (Murray and Brennan, 2012), bacterial blight by 94% in susceptible genotypes, and viral infections such as PSbMV and BLRV further reduce yield and seed quality, with PSbMV alone causing losses of up to 14% (Rubiales et al., 2019; Beck-Okins et al., 2022; van Leur et al., 2013). Therefore, deploying higher-yielding cultivars with improved yield stability and disease resilience remains the most cost-effective and practical approach for managing diseases in field pea.

Conventional breeding of field pea for yield and disease resistance relies on phenotypic selection across multiple environments, a process that takes 10–11 years to develop an improved breeding germplasm. This slow, labor-intensive, and costly method is limited by a narrow genetic base, often resulting in resistance that breaks down under disease pressure or with the emergence of a new strain of pathogen (Crossa et al., 2017; Lee et al., 2023; Rubiales et al., 2023; Parihar et al., 2025). Although worldwide field pea breeding programs have been in operation for many years, yield improvements have been limited (~0.93%) annually from 1994 to 2023 (FAO, 2025). Predicting how cultivars will perform across different environments remains a significant challenge due to complex genotype × environment (G × E) interactions and unpredictable growing conditions. With the emergence of genomic-assisted breeding, there is now an opportunity to accelerate and streamline the development of new varieties, thereby supporting global food security.

Genomic selection (GS) utilizes the relationship between genotype and phenotype within a population (i.e., the training population) to create a model for predicting the genotypic values of untested individuals (i.e., the testing population) for selection. GS efficiently captures the combined effect of all loci (i.e., both major and minor gene effects) using whole-genome markers and the genomic relationship among individuals for the genetic improvement of complex traits, such as yield, stress tolerance, and quality, to choose superior genotypes based on individuals’ genomic estimated breeding values (GEBVs) (Meuwissen et al., 2001; Heffner et al., 2009; Velazco et al., 2019; Escamilla et al., 2025). Empirical studies show that GS can increase genetic gains by shortening breeding cycles through rapid selection or improved selection efficiency with less field phenotyping (Rutkoski et al., 2015; Sallam et al., 2015; Tessema et al., 2020; Xu et al., 2020; Atanda et al., 2022; Dreisigacker et al., 2023). Rapid-cycle recurrent GS in the CIMMYT spring bread wheat breeding program reported a 12.3% genetic gain after three recombination cycles, increasing grain yield from 6.88–7.73 t ha^-1^ (Dreisigacker et al., 2023). GS can exceed conventional selection methods, allowing genetic gains 2–4 times higher in maize under drought conditions (Beyene et al., 2015, 2019). GS provided annual gains of 0.11–0.135 t ha^-1^ for maize under drought and waterlogging conditions (Das et al., 2020).

GS performance depends on several factors, including the heritability of the trait, training population size, marker density, statistical model, relatedness between the training and testing populations, accuracy of phenotyping, and the effective number of chromosome segments (Daetwyler et al., 2008; Heffner et al., 2011; Crossa et al., 2014; Brandariz and Bernardo, 2019; Xu et al., 2020). Standard models include RRBLUP and GBLUP, which assume the presence of many small-effect loci, and Bayesian models (i.e., BayesA, BayesB, BayesC, and LASSO) that allow for flexible marker effects (Meuwissen et al., 2001; Robertsen et al., 2019; Escamilla et al., 2025). Incorporating G × E, primarily through multi-environment GBLUP, improves prediction accuracy by accounting for genetic interactions across environments (Lopez-Cruz et al., 2015). Recent advances involve multi-trait, multi-environment models that enhance prediction by leveraging trait correlations and G × E interactions, particularly for low-heritability traits linked to those of high heritability (Burgueño et al., 2012; Jarquín et al., 2014; Cuevas et al., 2016; Millet et al., 2019; Gill et al., 2021).

Several studies have utilized GS in field pea to enhance traits such as grain yield, disease resistance, and seed quality, as well as to investigate factors influencing GS (Burstin et al., 2015; Annicchiarico et al., 2017; Carpenter et al., 2018; Annicchiarico et al., 2020, 2021; Bari et al., 2021; Atanda et al., 2022; Zhao et al., 2022; Castro-Urrea et al., 2023; Pecetti et al., 2023; Atanda and Bandillo, 2024; Saludares et al., 2024). These studies often rely on small sample sizes, limited phenotyping, and basic validation schemes, which may not fully capture the complexities of breeding programs. In contrast, breeding programs generate extensive phenotypic datasets that, although unbalanced, are well-suited to advanced multi-environment trial (MET) analyses and genomic prediction frameworks (Pandit et al., 2026). Integrating SNP-based genomic relationship matrices (GRM) within MET models enables more accurate estimation of genetic effects by capturing realized additive relationships arising from relatedness and Mendelian segregation (Hayes and Goddard, 2008; Keller et al., 2011; Rio et al., 2022). Consequently, there remains a clear need for empirical evaluations of GS under realistic breeding conditions, using large, heterogeneous populations evaluated across multiple years and locations, and validation strategies that closely mirror how GS is implemented in practice.

This study addresses the gap by leveraging historical phenotypic and genotypic data (2013–2022) from the National Field Pea Breeding Program at Agriculture Victoria Research, Australia. We assembled a reference population of 3,199 lines spanning multiple selection stages and evaluated it across diverse environments under field and controlled conditions. This is the first large-scale empirical study to assess GS for resistance to PSbMV, BLRV, and downy mildew in field pea. Using this extensive dataset, we developed and compared a suite of GS models, including single-trait GBLUP, multi-trait models, and those incorporating G × E interactions, to assess genomic prediction accuracy for grain yield and key biotic stress resistance traits in a large breeding population. We also tested cross-validation schemes designed to mimic practical breeding scenarios. Additionally, we discussed the implications and strategies for integrating GS into the routine breeding pipeline to enhance selection efficiency and accelerate genetic gains in field pea.

## 2 Materials and methods

### 2.1 Plant materials and phenotyping

This study used a comprehensive set of historical data from 2013 to 2022 for a reference population comprising 3,199 advanced breeding lines and commercial cultivars from the National Field Pea Breeding Program led by Agriculture Victoria Research, Victoria, Australia. The reference population was designed to represent the advanced breeding lines at a given time in the breeding program and consisted of all lines from various trial stages in the breeding cycles (i.e., advanced yield trials: stages 1 and 2, and stage 3, over the years from 2013 to 2022. Because the data originated from historical selection trials, the reference population is inherently unbalanced, reflecting the progressive advancement of superior lines through the breeding pipeline. The reference population was phenotyped for six traits: grain yield (GY), ascochyta blight (AB), pea seed-borne mosaic virus (PSbMV), bean leaf roll virus (BLRV), bacterial blight (BB), and downy mildew (DM), either in the field or in controlled environment conditions (**Supplementary Table 1**). GY, AB, BB, and BLRV were evaluated under field conditions, while the remaining biotic stresses were assessed in controlled environments.

The GY evaluation trials spanned from 2013 to 2022, covering eighteen sites across four Australian states: Victoria, South Australia, New South Wales, and Western Australia (**Supplementary Table 1**). The number of lines varied slightly each year, ranging from 327 to 851 from 2013 to 2022 (**Supplementary Figure 1A**). These trials employed a row-column design, with stage 1 lines partially replicated (20%) and stage 2 and 3 replicated twice. The trials included check varieties repeated across experiments to account for environmental variation and enhance genotype connectivity across trials, sites, and years. Lines were sown in plots measuring 1.25 × 5 m (6.25 m²), with a 0.25 m row spacing. Plot was harvested to estimate GY, which was then converted to t ha^-1^.

Among the biotic stresses, the lines were evaluated for resistance to AB, BB, and BLRV under field conditions, while PSbMV and DM were evaluated under controlled environment conditions (**Supplementary Table 1**). AB and BB were screened under natural infection in field trials during periods of heavy rainfall. The scoring was conducted twice, once at early flowering and once at early podding. The severity of the disease was quantified as the percentage of plant area affected. BB was estimated at the peak of disease infection during the pod-filling stage, with visual scores recorded on a 0–9 scale for each plot. BLRV was phenotyped at a field trial site in Breeza, New South Wales, from 2017 to 2022. A row-column design was implemented, and BLRV was inoculated through spreader rows. Disease assessment was conducted using the percentage (%) area of plant disease. PSbMV resistance was evaluated using a glasshouse (18–24 °C, 12/12-h light/dark) screening method with an augmented design that included repeated susceptible check (i.e., Kaspa), while the remaining lines were unreplicated. Seedlings were inoculated with the highly aggressive PSbMV-P1 pathotype according to the method reported by Joop et al. (2025). Two to three weeks after sowing, Tissue blot immunoassays (TBIA) were used to detect virus infections following the procedure described by (Kumari et al., 2022). TBIA provides rapid, reliable, and cost-effective results for a large number of samples (Freeman et al., 2013; Joop et al., 2025). Lines were scored on a 0–100 percentage scale, where 100% indicated that the disease affected 90–100% of plants in the pot. DM resistance was evaluated in a glasshouse (15–18 °C) using a 0–9 scale, with 9 indicating 100% plant infection.

### 2.2 Genotyping and SNP data calling

All lines were genotyped using the imputation-enabled multispecies pulse 30K SNP array, comprising 9,361 SNPs, as described in Gebremedhin et al. (2024). Briefly, DNA was extracted from 6 seeds per sample using a modified CTAB protocol (Tibbits et al., 2008). A total of 200 ng of pooled DNA per sample was used for the genotyping assay following the manufacturer’s protocols for the Infinium XT SNP bead chip array (Illumina Inc., San Diego, USA). Initial analysis was performed using GenomeStudio 2.0 Polyploid software (Illumina) using the manufacturer’s supplied crop-specific SNP manifest file. Theta and normalized R values were exported from GenomeStudio and used to call SNPs using the custom genotype-calling pipeline. Phasing and filling of missing data (~10%) were performed using Eagle/Beagle v4.1 (Browning and Browning, 2007, 2016) and aligned to the pea reference genome (Kreplak et al., 2019). Imputation with an accuracy of 95% at the whole-genome sequence level (8,201,627 SNPs) was performed using Minimac3 (Das et al., 2016) and whole-genome sequence (WGS)-based reference haplotypes. The reference panel for WGS-based imputation includes parents of breeding lines from the field pea breeding program, along with cultivars representing the available genetic diversity in field pea germplasm. SNP filtering thresholds were selected to balance marker informativeness and redundancy. Filtering was performed using vcftools (Danecek et al., 2011) and bcftools (Li, 2011) with a linkage disequilibrium (LD) sliding window size of 200 kbp, and R2 of pairwise LD < 0.99 and R2 of imputation > 0.8 were used to remove duplicate markers while retaining genome-wide coverage. SNPs were filtered to exclude those with a minor allele frequency (MAF < 0.05) and more than 20% missing values across lines. In addition, lines with more than 50% missing SNPs were discarded. After filtering, 39,565 high-quality genome-wide SNPs and 3,199 lines remained for further analysis. The distribution of SNPs across the seven field pea chromosomes was visualized using the *rMVP* package in R, showing genome-wide marker coverage and density (Yin et al., 2020) (**Supplementary Figure 2**). Genotypic data were formatted as a numeric matrix, and the kinship matrix (or GRM) was computed using the *GAPIT* package (Wang and Zhang, 2021). Population structure was assessed via principal component analysis (PCA) of the GRM, with the variance explained by the top PCs visualized in scree plots. K-means clustering was applied to the first three PCs to identify genetic groups, with the optimal number of clusters determined using the elbow method. Cluster membership was visualized in 2D and 3D PCA plots (**Supplementary Figure 3**). All analyses were performed in R using the tidyverse, gplots, plotly (Sievert, 2020), and factoextra. Due to the usage of proprietary breeding material in this study, the genotype and SNP names have been de-identified in the genotyping data file (**Supplementary File 1**).

### 2.3 Phenotypic data analysis

All the phenotypic analyses were conducted using ASReml-R v4.1 (Butler et al., 2017). Prior to modeling, data were subjected to standard quality-control procedures to improve phenotypic reliability, including outlier removal, correction of data errors, and assessment of distributional assumptions. Moreover, trials with low data quality were excluded to reduce noise in subsequent analyses: trials with broad-sense heritability (*H²*; discussed later) < 0.2 were removed for field-measured traits (GYD, AB, BB, and BLRV), while a lower threshold (H² < 0.1) was applied to traits assessed under controlled conditions (DM and PSbMV), reflecting differences in experimental precision, replication, and trait genetic architecture.

For the analysis, a two-stage mixed-model approach was employed to evaluate the phenotypic data for all traits (Piepho et al., 2012; Smith and Cullis, 2018). This approach was chosen to address the significant variability in experimental designs across different trials. A stage-wise analytical framework is especially effective for breeding datasets that are unbalanced, feature diverse design structures at various trial stages, and include numerous experiments. By analyzing trials separately in the initial stage, this method ensures accurate modeling of trial-specific effects while avoiding the computational and convergence difficulties that come with fitting a single, large-scale model to all trials at once (Damesa et al., 2017).

In the first stage, we used lines as a fixed effect to extract the adjusted means per trial, as best linear unbiased estimates (BLUEs), and residuals were computed for each trial, accounting for trial-specific design effects to ensure consistent and independent estimation of genotype performance within each environment (Equation 1). This approach helps avoid shrinkage of genotype effects, which is desirable when BLUEs are subsequently used as input for genomic prediction models (Piepho et al., 2008). To account for spatial variation in the field layout, the phenotypic response was modeled using the following linear model,

(1) y = μ+Xβ +e

where ***y*** is the n×1 vector of phenotypic observations for each plot, **X** is the design matrix for fixed effects, and β is the corresponding vector of fixed-effect parameters, including the overall mean (*μ*), genotype effects ( $G_{k}$), and block effects ( $B_{j}$). The residual term **ℯ** accounts for spatially correlated random errors. Different residual variances were allowed across trials, while a common spatial correlation structure was assumed. Each trial was treated as a rectangular array of rows (r) and columns (c), and residuals were assumed to follow a multivariate normal distribution e∼N(0,R), with the residual covariance structure defined as $R={\text{ σ}}_{\text{e}}^{2}\;(\Sigma_{\text{c}}(\rho_{c})\;⊗\;\Sigma_{\text{r}}(\rho_{r}))$. The $\;\sigma_{e}^{2}$ is the residual variance, whereas $\Sigma_{\text{c}}(\rho_{c})$ and $\;\Sigma_{\text{r}}(\rho_{r})$ represents the first-order auto-regressive correlation matrices for the column and row directions of the field layout, respectively, and $\rho_{c}$ and $\rho_{r}$ are the auto-correlation parameters. The Kronecker product ⊗ represents a separable auto-regressive (i.e., AR1 × AR1) spatial structure, which effectively captures gradual spatial variation in regularly arranged breeding trials and is standard practice in MET field trials (Gilmour et al., 1997; Andrade et al., 2020; Bernardeli et al., 2021). All models were fitted using restricted maximum likelihood (REML) with the Average Information (AI) algorithm implemented in ASReml. Model convergence was evaluated based on stabilization of the log-likelihood and variance component estimates, using ASReml’s default convergence criteria. Where necessary, the maximum number of iterations was increased to 100 to ensure stable convergence. The resulting BLUE phenotypes were used as input to the genomic prediction models, discussed later.

Broad sense heritability ( $H^{2}$) for each trial was calculated according to the method reported by (Cullis et al., 2006) (Equation 2).

(2) $$H_{Cullis}^{2}=1-\frac{{\bar{V}}_{BLUP}}{2\sigma_{g}^{2}\;}$$

where ${\bar{V}}_{BLUP}$ is the mean-variance difference of two lines based on BLUPs and $\sigma_{g}^{2}$ is the variance of lines.

### 2.4 Genetic correlation for grain yield between environments

A bivariate model was employed to investigate the G × E interaction, combining all trials conducted in a single year at a single site into a single environment (i.e., site × year combination), considering that within a site in a given year, environmental conditions (soil, management, climate) are relatively homogeneous (Hussain et al., 2022; Miller et al., 2023). Best linear unbiased predictions (BLUPs) were estimated by fitting genotype and G × E interaction effects as random effects in the model (Equation 3). The variance structure was assumed to be heterogeneous across environments, modelled using the “Unstructured (US)” term.

(3) $$y\;=\;\mu +X\beta +Z_{g}a+e$$

where, yis the vector of observed responses (the adjusted BLUEs from Equation 1), the μ represents the overall mean across all trials, X is the design matrix for fixed effects, including the environment (i.e., site × year), β is the vector of fixed effects. $Z_{\text{g}}$ is the design matrix for random genetic effects, with $a∼N(0,G_{A}⊗G_{b}^{2})$, where G is the genomic relationship matrix (GRM), ⊗ denotes the Kronecker product and $G_{A}=[\begin{array}{cc} \sigma_{a_{1}}^{2} & \sigma_{a_{12}}\\ \sigma_{a_{21}} & \sigma_{a_{2}}^{2} \end{array}]$, where $\sigma_{a_{1}}^{2}$ is the additive genetic variance for environment 1, $\sigma_{a_{2}}^{2}$ is the additive genetic variance for environment 2, $\sigma_{a_{12}}$ and $\sigma_{a_{12}}=\sigma_{a_{21}}$, the additive genetic covariance between environment 1 and environment 2. $e_{ij}$ is the vector of residuals with e∼N(0,I⊗R), I is the identity matrix, $R=[\begin{array}{cc} \sigma_{e_{1}}^{2} & 0\\ 0 & \sigma_{e_{2}}^{2} \end{array}]$, where $\sigma_{e_{1}}^{2}$ and $\sigma_{e_{2}}^{2}$ are the residual variances for Environment 1 and Environment 2. The genetic correlation between traits was calculated as ${\text{ r}}_{\text{g}}=\frac{{\text{σ}}_{{\text{a}}_{12}}}{\sqrt{{\text{σ}}_{{\text{a}}_{1}}^{2}\times {\text{ σ}}_{{\text{a}}_{2}}^{2}}}$.

The narrow sense heritability (*h*^2^) for each trait was estimated as the proportion of genetic variance explained by additive genetic effects relative to the total phenotypic variance (Equation 4).

(4) $$h^{2}=\frac{\sigma_{a}^{2}}{(\sigma_{a}^{2}\;+\;\sigma_{e}^{2})\;}$$

where, $\sigma_{a}^{2}$ is the additive genetic variance, $\sigma_{e}^{2}$ is the residual variance.

### 2.5 Clustering of environments for grain yield

The environments (i.e., site × year combination) were grouped into clusters to identify regions with similar patterns of genotypic performance, following the method described by Lin et al. (2021). Agglomerative hierarchical clustering was applied to a correlation matrix of genotype BLUEs, representing pairwise similarity in genotype responses across environments (compiled in section 2.4). The hierarchical tree was constructed using the “*hclust”* function in R, applying the Ward method, which merges environments to minimize the within-cluster sum of squares, producing compact, interpretable clusters with similar genotype rankings. The optimal number of clusters was objectively determined using the Kelley-Gardner-Sutcliffe (KGS) penalty function (“*kgs”* function in R) implemented in the *maptree* package (White and Gramacy, 2012). The KGS metric balances within-cluster homogeneity with model complexity by penalizing overly large cluster counts. The penalty curve exhibited a clear minimum at *k* = 5 (**Supplementary Figure 4**), indicating that five clusters are the most parsimonious and stable partition. This criterion avoids subjective selection and enhances reproducibility.

### 2.6 Genetic correlation between traits

A slightly modified version of the model in Equation 3 was also employed to estimate genetic correlations among traits. In this equation, the $\sigma_{a_{1}}^{2}$ and $\sigma_{a_{2}}^{2}$represent the additive genetic variance for traits 1 and 2, respectively, while $\sigma_{e_{1}}^{2}$and $\sigma_{e_{2}}^{2}$ denote the corresponding residual variances for traits.

### 2.7 Genomic prediction models

GBLUP genomic prediction models were fitted with and without G × E interactions (i.e., GBLUP G × E and GBLUP non-G × E) to calculate GEBVs for GY, AB, BB, and BLRV, while only the model without G × E was applied to PSbMV and DM, as these traits were measured only in a controlled environment, where G × E interactions cannot be estimated. The GBLUP non-G × E model includes a single random genetic effect as follows:

(5) $$y\;=\mu +\;X\beta \;+\;Z_{g}g+\;e\;$$

Where y is the vector of observed responses (adjusted BLUEs), the μ represents the overall mean across all trials, X is the design matrix for fixed effects, including trial, site, and year. and β is the fixed effect coefficient. $Z_{\text{g}}$ is the design matrix for random genetic effects. g is the random effect, with $g∼N(0,\sigma_{g}^{2}G)$, where G is the additive genomic relationship matrix and ${\text{ σ}}_{\text{g}}^{2}$ is the genetic variance. e is the vector of residuals with $e∼N(0,I\sigma_{e}^{2}),$ I is the identity matrix, and the ${\text{ σ}}_{\text{e}}^{2}$ is the residual variance. By exploiting marker-derived relationships among individuals, genomic information enables the modelling of genetic performance across heterogeneous and unbalanced environments, reducing reliance on extensive genotype replication and facilitating the prediction of genotype performance in previously untested environments (Burgueño et al., 2012; Guo et al., 2013; Endelman et al., 2014; Ankamah-Yeboah et al., 2020; Jarquin et al., 2020). Therefore, the additive genomic relationship matrix was calculated using VanRaden’s method for all genotyped lines, as shown in **Supplementary Figure 3A**.

In the GBLUP G × E model, the performance of lines across multiple environments was modeled using a multi-environment linear mixed model (Equation 6):

(6) $$y=\mu +X\beta +Z_{1}u_{1}+\;Z_{2}u_{2}+e$$

Where, y is the vector of observed trait data for all lines and environments. The μ represents overall mean across all environments, same as Equation 5. $Z_{2}$ is the design matrix for G × E interaction effects, modeled using a cross-factor analytic (XFA) structure. In this model, $u_{2}∼N(0,\Sigma_{G\times E}),$ where the co-variance matrix $\Sigma_{\text{G}\times \text{E}}$ was modeled as a factor analytic structure: $\Sigma_{G\times E}=LL^{T}+D$, where L is a matrix of loadings (latent factors) for G × E patterns, and D is a diagonal matrix of environment-specific variances. The FA variance models with *k* = 1, 2, and 3 were evaluated. The FA (*k* = 1) model was selected because it provided a parsimonious and stable representation of the G × E structure, whereas higher-order models showed no substantial improvement in fit and increased parameter complexity. This choice is consistent with previous multi-environmental GS studies and avoids over-parameterization given the level of connectedness among environments.  e is the residual errors are modeled as $e∼N(0,I\sigma_{e}^{2}),$ where σe2 is the residual variance and I is the identity matrix.

As described in Section 2.4, Equation 3 was also used for bivariate GBLUP to predict GEBVs for GY, AB, BB, and BLRV. In Equation 3, where $y=[\begin{array}{c} \text{y}1\\ \text{y}2 \end{array}]$ is the vector of observed responses (adjusted BLUEs from Equation 1) for the two traits (i.e., trait 1 and trait 2). X is the design matrix for fixed effects, β is the vector of fixed effects. ***Z*** is the design matrix for random genetic effects (described above), $R_{0}=[\begin{array}{cc} {\text{σ}}_{{\text{e}}_{1}}^{2} & {\text{σ}}_{{\text{e}}_{12}}\\ {\text{σ}}_{{\text{e}}_{12}} & {\text{σ}}_{{\text{e}}_{2}}^{2} \end{array}]$ where ${\text{σ}}_{{\text{e}}_{1}}^{2}$ and ${\text{σ}}_{{\text{e}}_{2}}^{2}$ are the residual variances for trait 1 and trait 2, respectively. ${\text{σ}}_{{\text{e}}_{12}}$ is the residual covariance between trait 1 and trait 2, and I is the identity matrix as described in the section above.

### 2.8 Cross-validation and genomic prediction accuracy

We employed two cross-validation strategies to evaluate model performance: five-fold cross-validation and leave-one-out by year (LYO-CV), both aimed at estimating trait breeding values. In a five-fold cross-validation, all individuals were randomly divided into five groups of equal size, with the BLUEs of the four groups (≈80% of the population) used as the training set. The remaining group served as a test set (≈20% of the population), and genomic breeding values of this group were predicted. This procedure was repeated until all subsets had served as validation sets. The whole process was repeated 10 times. Prediction accuracy was calculated as the Pearson correlation between observed BLUEs and predicted GEBVs, and results from all folds were combined to calculate the mean genomic prediction accuracy and its standard error.

To more closely mimic forward prediction across breeding cycles, a leave-one-year-out cross-validation (LYO-CV) scheme was implemented. In LYO-CV, all lines evaluated in a single year were assigned to the validation population, while phenotypic and genotypic data from the remaining (n − 1) years were used for training the prediction model. This process was repeated iteratively for each year, and prediction accuracy was calculated as the correlation between BLUEs from the validation year and the corresponding GEBVs. This design explicitly accounts for temporal structure in the data and reflects practical deployment of GS, where selection candidates often belong to future breeding cycles. Two LYO-CV scenarios were evaluated:

1. CV0 – prediction of tested lines in untested environments, where lines in the validation year had phenotypic records in other years and thus also appeared in the training set. This scenario benefits from higher genetic connectedness but may yield optimistic accuracy estimates due to information leakage arising from repeated observations of the same lines across years.
2. CV1 – prediction of untested lines in untested environments, where lines in the validation year were entirely absent from the training population. This scenario represents true forward prediction, mimicking early-generation selection decisions in breeding programs where newly developed lines lack prior phenotypic information. Uneven genotype representation across years was mitigated by iterative LYO-CV across all years, though we acknowledge that sparsely represented years may still influence accuracy estimates.

Within-cluster validation for GY was performed using a non-G × E GBLUP model to estimate cluster-specific prediction performance. This conservative approach avoids over-parameterization, given the smaller number of environments per cluster, while providing meaningful guidance for early-stage selection. All analyses were conducted in R with fixed random seeds to ensure reproducibility, and identical model settings were applied across iterations. Mean prediction accuracy and standard deviation were reported for all cross validations.

## 3 Results

### 3.1 Phenotypes and heritability

BLUEs were calculated for each trial and trait, and the statistical summary is shown in **Table 1**. A total of 2,519 lines were assessed, resulting in an average yield of 2.28 t ha^-^¹ (SD = 0.98; see **Table 1**) across 197 trials spanning 7 years (2013–2022; except 2015, 2017, and 2018) and 18 sites (**Supplementary Table 1**). For GY, **Supplementary File S1A** details genotype counts per year and the number of lines unique to each year, indicating ongoing introduction of new lines and their transition to different stages or out of the breeding program. A connectivity matrix illustrating shared lines between years (**Supplementary Figure 1B**) showed uneven overlap: some year pairs shared over 300 lines, while others shared fewer than 10. For GY and disease resistance traits, connectivity, defined as the number of shared lines between trials, varied from 2 to 500, highlighting the unbalanced nature of the dataset (**Supplementary Table 2**).

**Table 1:** Summary of single-stage BLUEs (per trial) for key field pea traits, including the number of BLUEs, sites, lines, trials, and environments (site × year combinations), mean ± standard deviation (SD) of BLUEs, broad-sense heritability (*H*²; mean ± standard error, SE and range), and narrow-sense heritability and SE (*h*² ± SE).

Traits^*^	No. of BLUEs	No. of sites	No. of lines	No. of trials	No. of env^**^	Means ± SD BLUEs	*H^2^* Mean ± SE	*H^2^* range	*h^2^* ± SE
GY	26327	18	2519	197	74	2.28 ± 0.98	0.67 ± 0.12	0.2 – 0.97	0.48 ± 0.03
AB	3062	6	1742	15	6	65.46 ± 26.37	0.59 ± 0.04	0.27 – 0.83	0.23 ± 0.03
BB	1886	3	1243	6	4	3.72 ± 1.02	0.46 ± 0.1	0.18 – 0.89	0.23 ± 0.04
PSbMV^***^	573	–	523	5	–	71.03 ± 37.29	0.49 ± 0.11	0.16 – 0.67	0.52 ± 0.06
BLRV	750	1	633	6	6	25.03 ± 21.32	0.69 ± 0.06	0.4 – 0.88	0.46 ± 0.06
DM^***^	718	–	615	4	–	3.65 ± 1.36	0.38 ± 0.08	0.12 – 0.51	0.21 ± 0.05

^*^ Grain yield, GY; ascochyta blight, AB; pea seed-borne mosaic virus, PSbMV; bean leaf roll virus, BLRV; bacterial blight, BB; and downy mildew, DM.

^**^ The number of environments refers to the site × year combinations.

^***^ Traits assessed in a controlled condition; PSbMV experiments in 2020 and 2022 lacked replication.

The broad-sense heritability (*H^2^*) for GY and biotic stress tolerance traits varied widely, ranging from 0.12 to 0.97, and only three trials had low heritability (< 0.2), including PHO17, P2HO17, and PTM19 for BB, DM, and PSBMV, respectively (**Supplementary Table 3**). Among the traits, BB had the lowest mean heritability (*H^2^* = 0.46 ± 0.1), while BLRV exhibited the highest mean heritability (*H^2^* = 0.69 ± 0.06; **Table 1**). Lower heritability in some environments was associated with higher residual variance and coefficients of variation (CV), reflecting increased environmental noise (**Supplementary Table 3**). The narrow-sense heritability (*h^2^*) of GY was moderate (0.48 ± 0.03), while BB and AB showed lower narrow-sense heritability estimates of 0.23 ± 0.04 and 0.23 ± 0.03, respectively. PSbMV exhibited the highest narrow-sense heritability at 0.52 ± 0.06, followed by BLRV with an estimate of 0.46 ± 0.06 (**Table 1**). For traits evaluated under controlled-environment conditions, heritability estimates were generally higher and less variable, reflecting reduced environmental noise.

### 3.2 Genetic correlation within and between traits

**Figure 1:**
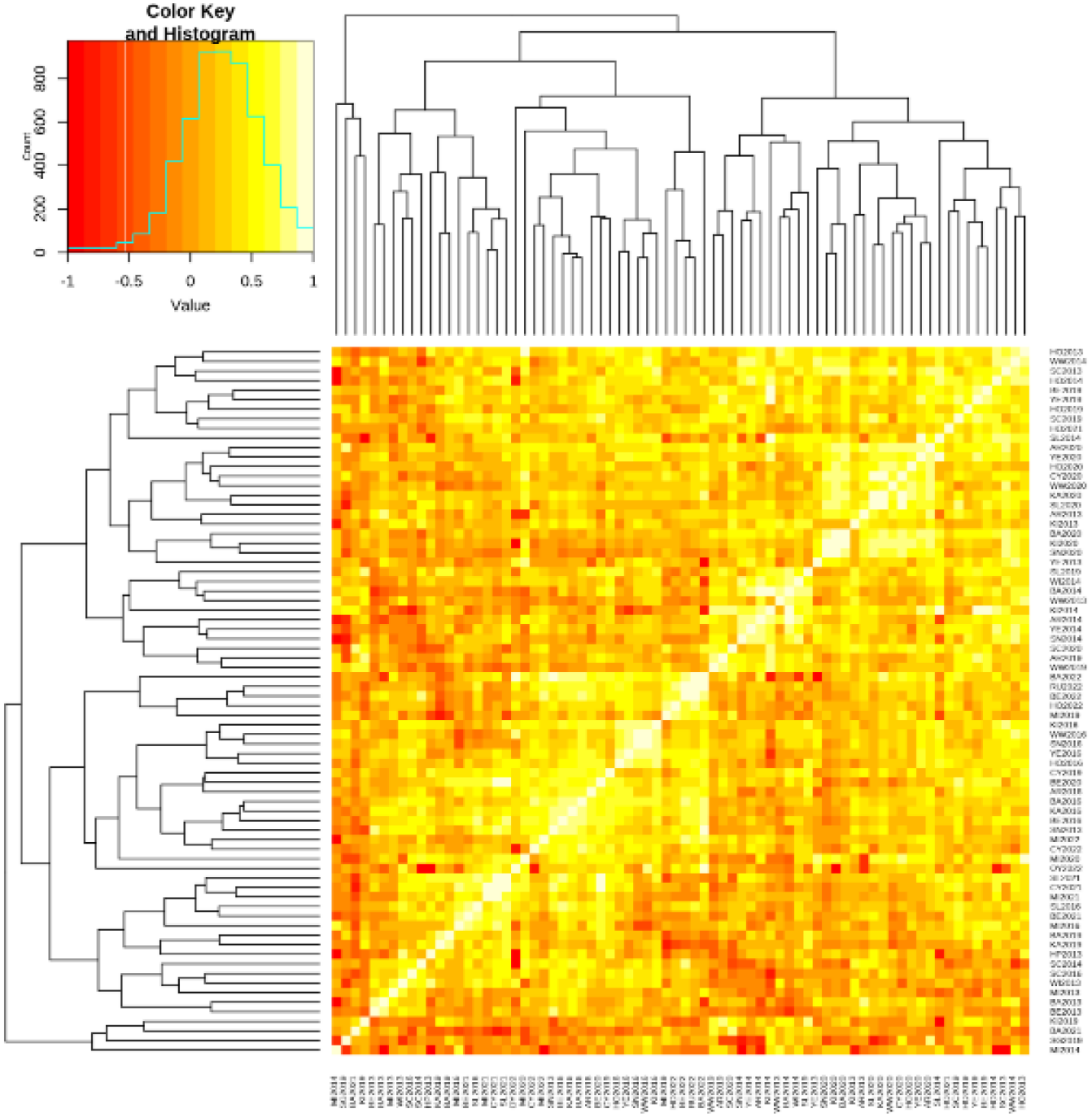
Pairwise genetic correlation analysis of grain yield (GY) across different environments (site × year combinations), illustrating the extent and heterogeneity of genotype × environment interaction. The intensity of the color indicates the strength and direction of correlations between lines in various environments.

Genetic correlations among different environments (i.e., site × year combinations) were assessed for field-measured traits, including GY, AB, BB, and BLRV (**Supplementary Table 4**). The number of environments evaluated for each trait is presented in **Table 2**, along with detailed genetic correlations available in **Supplementary Table 4**. For GY, genetic correlations between paired environments (n = 2,701) measure the extent of G × E interaction (**Figure 1**) and range from −0.99 to 0.99, indicating substantial G × E interaction. A majority (87.93%, n = 2,375) of environment combinations exhibited low correlations (r_g_ < 0.6), while n = 326 (12%) environments showed moderate to high genetic correlations (r_g_ ≥ 0.6) (**Figure 1**; **Supplementary Table 4**), reflecting limited consistency in genotypic performance across environments. Extreme values often involve pairs with low genetic relatedness or contrasting conditions and are unlikely to reflect broadly repeatable responses. The focus was on overall patterns and proportions rather than on extremes. The dominance of weak correlations in GY suggests substantial G × E crossover, with genotypic rankings often environment-specific, reducing performance predictability.

**Table 2:** Summary statistics of environment clusters used in the genomic prediction analysis for grain yield (GY; t ha^-^1).

Clusters	No. of lines	No. of BLUEs	No. of trials	No. of stage	No. of year	No. of sites	No. of env	Mean GY ± SD	Broad-sense heritability (*H*^2^) range	Mean Broad-sense heritability (*H*^2^) ± SE
1	1725	8013	68	3	5	12	23	2.47 ± 1.02	0.29 – 0.97	0.65 ± 0.02
2	987	2062	30	3	5	7	9	1.99 ± 0.91	0.31 – 0.94	0.68 ± 0.04
3	1184	3767	37	3	5	9	13	1.60 ± 0.74	0.44 – 0.95	0.75 ± 0.02
4	1866	8590	40	3	5	12	20	2.69 ± 0.92	0.3 – 0.9	0.68 ± 0.02
5	1776	3895	22	3	4	7	9	1.79 ± 0.63	0.2 – 0.83	0.56 ± 0.03
Total	2519	26327	197	3	7	18	74	2.28 ± 0.98		

For each cluster, the table reports the number of lines evaluated, the number of best linear unbiased estimates (BLUEs), mean grain yield (± standard deviation, SD), and the number of trials, breeding stages, years, sites, environments (site × year combinations), and broad-sense heritability (*H*^2^) range per trial for each cluster. The total row summarizes the overall dataset across all clusters.

For disease traits, the genetic correlation ranged from −0.18 to 0.81 for AB (**Supplementary Table 4**). For BB, the correlations ranged from −0.24 to 0.72, while for BLRV, r_g_ spanned −0.96 to 1.0. Genetic correlations for controlled-environment traits (PSbMV and DM) between trials were estimated from fewer trials and showed greater variability. Similarly, for PSbMV, genetic correlations between trials ranged from −0.96 to 0.67, and for DM, the genetic correlation (r_g_) values ranged from −0.03 to 0.98. These estimates should therefore be interpreted cautiously, as reduced trial numbers limit statistical power and increase sampling variance.

**Figure 2:**
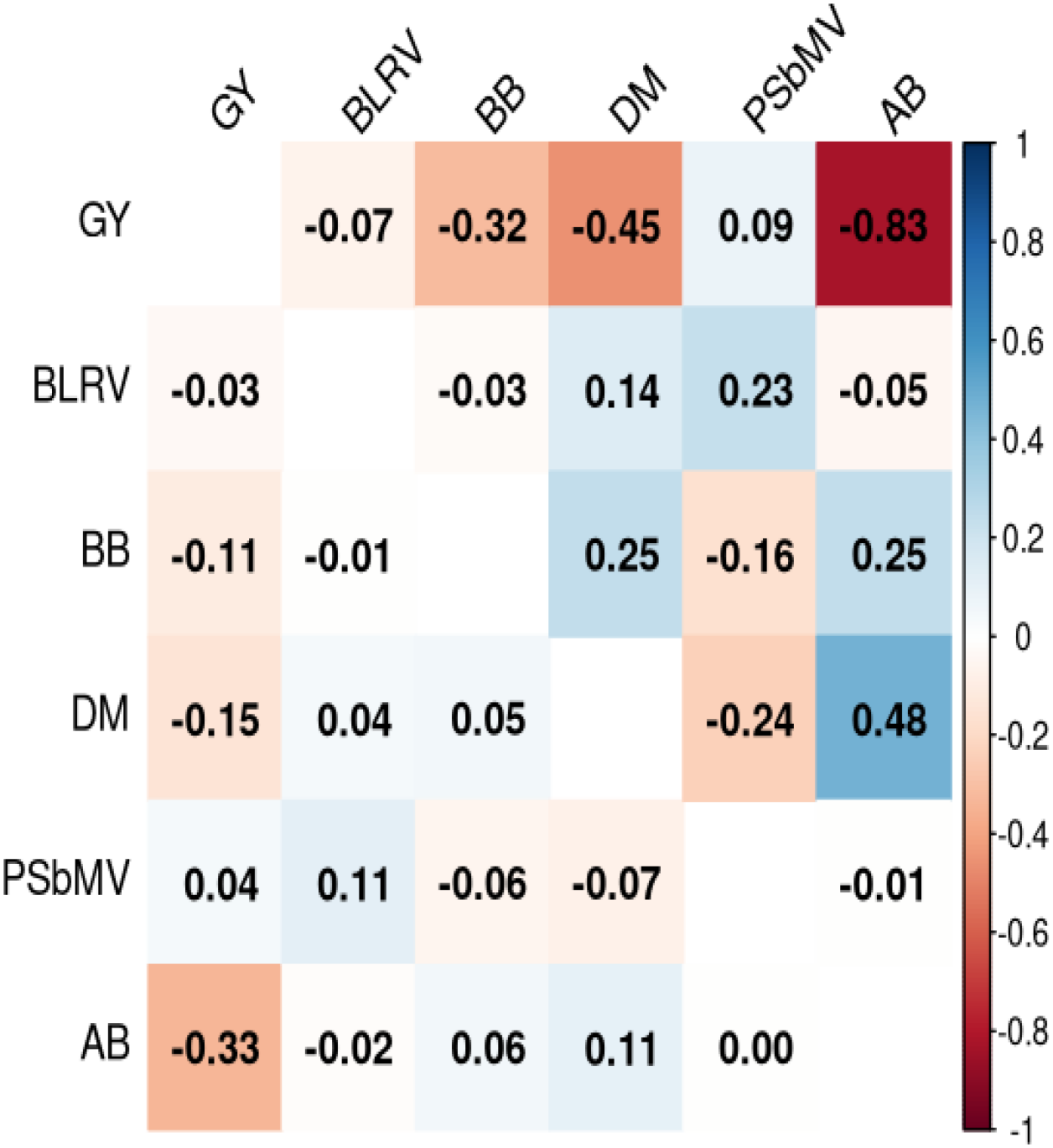
Pairwise genetic correlations (upper diagonal) and phenotypic correlations (lower diagonal) among grain yield (GY) and disease traits, including ascochyta blight (AB), pea seed-borne mosaic virus (PSbMV), bean leaf roll virus (BLRV), bacterial blight (BB), and downy mildew (DM).

The genetic and phenotypic correlations (r_g_ and r_p_) between traits were also assessed, where GY showed a strong negative genetic correlation with AB (r_g_ = −0.83 ± 0.04), BB (r_g_= −0.32 ± 0.01), and DM (r_g_= −0.45 ± 0.12), suggesting that an increase in severity in AB, BB, or DM is associated with a reduction in GY (**Figure 2**). Similarly, negative phenotypic correlations were observed between GY and AB (r_p_ = −0.33 ± 0.03) and between GY and BB (r_p_= −0.11 ± 0.04). BB exhibited moderate positive genetic correlations with DM (r_g_ = 0.25 ± 0.16) and AB (r_g_ = 0.25 ± 0.11). Moreover, PSbMV and BLRV exhibited weak genetic (r_g_ = 0.09 ± 0.01; r_g_ = 0.07 ± 0.04) and phenotypic (r_p_= 0.04 ± 0.05; r_p_ = 0.032 ± 0.05) correlations with GY, indicating limited direct association with GY under the evaluated conditions (**Figure 2**).

Overall, the genetic correlation structure highlights pronounced environmental heterogeneity for GY and moderate genetic associations between GY and disease traits, providing a basis for subsequent analyses of G × E modelling, environment clustering, and multi-trait genomic prediction.

### 3.3 Clustering of environments for grain yield

**Figure 3:**
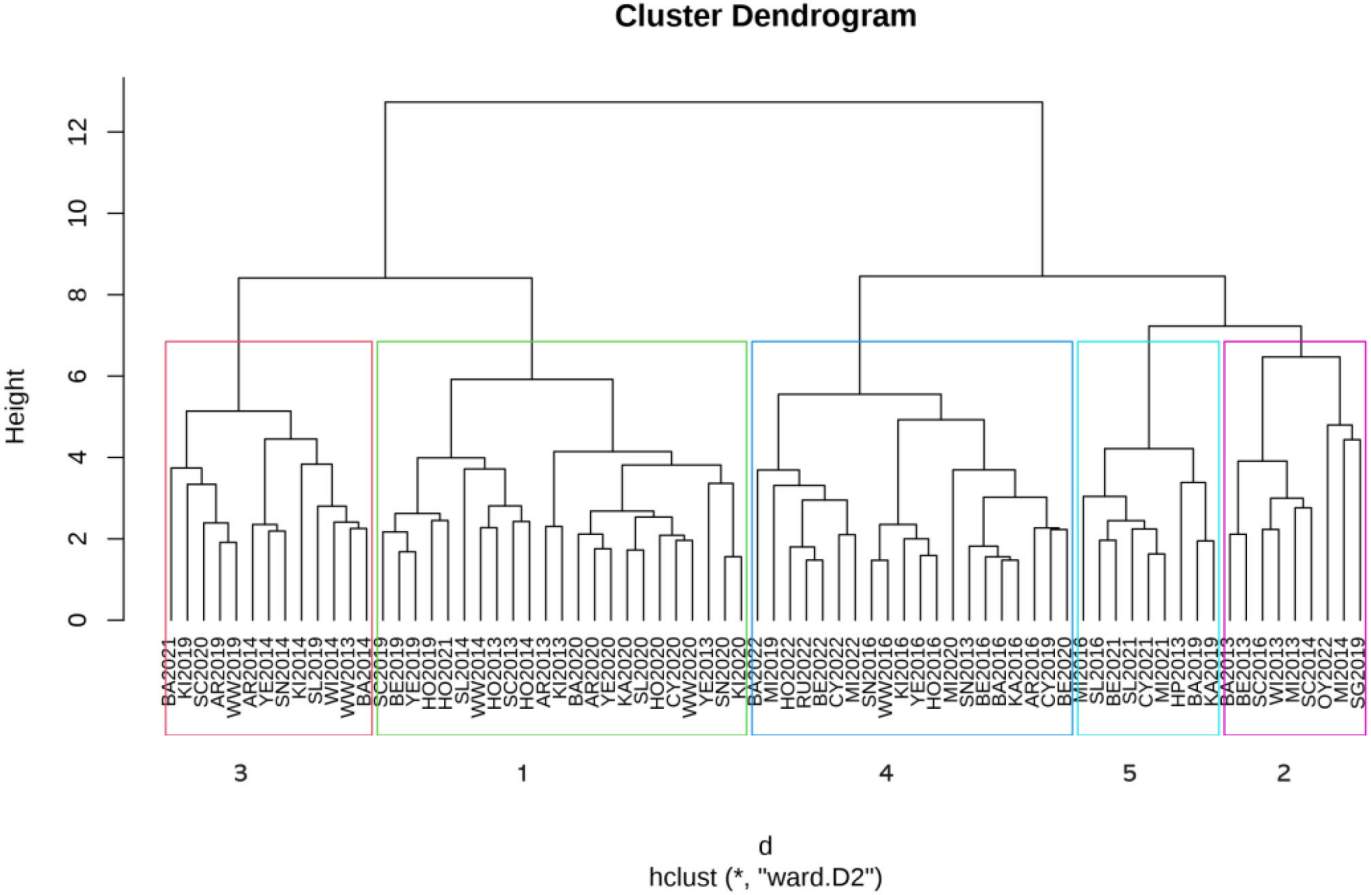
Hierarchical clustering of grain yield (GY) environments based on pairwise genetic correlations, with clusters highlighted (numbered) by colored rectangles.

We conducted a clustering analysis across 74 environments (i.e., site × year combinations), which included 197 trials examining GY (**Figure 3**). The analysis identified five distinct clusters (**Figure 3**; **Supplementary Figure 4**). Summary statistics of the environmental clusters used in the genomic prediction analysis for GY were presented in **Table 2**. Clusters were unbalanced in size and genetic composition, reflecting differences in trial representation, breeding stage coverage, and genotype overlap, with pairs of clusters sharing 605−1866 lines (**Table 2**; **Supplementary Figure 5**). Cluster 4 emerged as the largest, with 1,866 individuals in the training population, and the highest mean GY of 2.69 ± 0.92 t ha^-1^ across 20 environments. Cluster 1 similarly encompassed a large training population (1,725 lines) across 23 environments, with a mean GY of 2.47 ± 1.02 t ha^-^¹. In contrast, Cluster 3 included fewer environments (n = 13) and sites (n = 9) and showed the lowest mean GY (1.60 ± 0.74 t ha^-^¹), despite moderate to high trial-level heritability (mean H² = 0.75 ± 0.02). Cluster 5 was characterized by lower mean GY (1.79 ± 0.63 t ha^-^¹) and the lowest average heritability (0.56 ± 0.03), whereas Cluster 2 showed intermediate mean GY (1.99 ± 0.91 t ha^-^¹) and heritability estimates (**Supplementary Table 5**). Across clusters, broad-sense heritability for GY varied widely among trials (*H²* = 0.20–0.97), highlighting substantial heterogeneity across environments (**Table 2**).

### 3.4 Genomic prediction accuracies for grain yield

Genomic prediction accuracies were evaluated for all traits, and the results were summarized in **Table 3**. While the more challenging LYO cross-validation with GBLUP, excluding G × E effects, showed moderate accuracies (0.33 and 0.37; **Table 3**) with corresponding standard errors of 0.09 and 0.06, incorporating G × E effects led to slight but consistent improvements, increasing accuracies to 0.34 (CV1, Standard error-SE = 0.09) and 0.38 (CV0, SE = 0.07) across different scenarios (**Table 3**).

**Table 3:** Mean genomic prediction accuracies (± standard deviation) for all traits using two cross-validation methods: five-fold and leave-one-out by year (LYO) based validation.

Trait		Leave-one-out by year			
	k-fold	GBLUP non-G × E		GBLUP G × E	
	Five-fold	CV0	CV1	CV0	CV1
GY	0.56 ± 0.003^**^	0.37 ± 0.15	0.33 ± 0.19	0.38 ± 0.19	0.34 ± 0.2
AB	0.41 ± 0.005	0.26 ± 0.08	0.22 ± 0.09	0.28 ± 0.06	0.25 ± 0.08
BB	0.31 ± 0.006	0.34 ± 0.03	0.28 ± 0.08	0.34 ± 0.05	0.31 ± 0.09
PSbMV^*^	0.42 ± 0.007	0.72 ± 0.09	0.68 ± 0.12	–	–
BLRV	0.42 ± 0.007	0.53 ± 0.09	0.49 ± 0.09	0.52 ± 0.09	0.49 ± 0.1
DM^*^	0.21 ± 0.008	0.29 ± 0.06	0.26 ± 0.04	–	–

The LYO method is evaluated under CV0 (tested lines in untested environments) and CV1 (untested lines in untested environments). Predictions are assessed using GBLUP non-G × E and GBLUP G × E.

^*^ Traits assessed in a controlled environment do not include G × E interactions.

^**^ Standard error (SE) calculated for five-fold cross-validation.

The prediction accuracies within each cluster were analyzed using the GBLUP non-G × E model, with LYO cross-validation under two scenarios, CV0 and CV1, and the results are presented in **Table 4**. In the CV0 scenario, where tested lines were evaluated in untested environments, prediction accuracies ranged from 0.21 ± 0.05 to 0.45 ± 0.14 across clusters. Cluster 1 exhibited the highest prediction accuracy (0.45 ± 0.14), followed by Cluster 4 (0.44 ± 0.11), whereas Cluster 5 had the lowest accuracy (0.21 ± 0.05). Under the CV1 scenario, which assessed untested lines in untested environments, the prediction accuracies were generally lower, ranging from 0.10 ± 0.09 to 0.37 ± 0.14. Cluster 4 demonstrated relatively high accuracy (0.37 ± 0.14), whereas Cluster 3 exhibited the lowest accuracy (0.10 ± 0.09; **Table 4**).

**Table 4:** Mean genomic prediction accuracies (± standard deviation; SD) using GBLUP non-G × E for GY clusters under two cross-validation scenarios: CV0 (tested lines in untested environments) and CV1 (untested lines in untested environments).

Clusters	Mean prediction accuracy ± SD	
	CV0	CV1
1	0.45 ± 0.14	0.36 ± 0.23
2	0.23 ± 0.16	0.26 ± 0.18
3	0.24 ± 0.07	0.10 ± 0.09
4	0.44 ± 0.11	0.37 ± 0.14
5	0.21 ± 0.05	0.14 ± 0.05

Based on low to moderate genetic correlations among traits assessed under field conditions (**Figure 2**), AB, BB, and BLRV were used as secondary traits to predict GY using a bivariate GBLUP model with five-fold random cross-validation. The mean genomic prediction accuracy for GY was 0.59 ± 0.02 in the GY-AB, 0.60 ± 0.02 in the GY-BLRV model, and 0.6 ± 0.03 in the GY-BB model.

### 3.5 Genomic prediction accuracies for biotic stress resistance

For biotic stress resistance traits, the five-fold cross-validation, prediction accuracies ranged from 0.21 ± 0.008 for DM to 0.42 ± 0.007 for both PSbMV and BLRV (**Table 3**). For the GBLUP model without G × E interactions, PSbMV demonstrated the highest prediction accuracy in LYO CV0 (0.72 ± 0.09), and the lowest for DM (0.21 ± 0.08). Mean genomic prediction accuracies in CV1 declined, ranging from 0.26 ± 0.04 (DM) to 0.68 ± 0.12 (PSbMV). When considering GBLUP G × E models, which accounted for G × E interactions, only AB, BB, and BLRV traits were assessed. However, the GBLUP G × E prediction accuracies were either similar to or lower for the AB, BB, and BLRV traits for the GBLUP non-G × E model. Specifically, prediction accuracies were 0.26 ± 0.08 (CV0) and 0.25 ± 0.08 (CV1) for AB, 0.34 ± 0.03 (CV0) and 0.31 ± 0.09 (CV1) for BB, and 0.52 ± 0.09 (CV0) and 0.49 ± 0.1 (CV1) for BLRV (**Table 3**).

## 4 Discussion

### 4.1 Reference population design and phenotypic data structure for GS in the breeding program

This study evaluated GS in field pea using a reference population of 3,199 advanced breeding lines, and cultivars phenotyped between 2013 and 2022 within the Australian National Field Pea Breeding Program. Although its size and composition were constrained by available phenotyping and genotyping resources, the reference set captures the genetic diversity, allele frequencies, and selection history, making it highly relevant for GS in a breeding program. Since 2017, GS has been progressively integrated into the public breeding pipeline, demonstrating its practical utility under operational conditions.

Analysis of the GRM and PCA (**Supplementary Figure 3**) revealed a largely interconnected population structured into four main clusters, with significant overlap among lines from different years. This pattern involves recycling elite germplasm through biparental and complex crossing schemes, maintaining genetic connectivity across breeding cycles (Piepho et al., 2008; Khanna et al., 2022a, b). Such relatedness is a key determinant of prediction accuracy and becomes particularly important when historical datasets spanning multiple years are used for model training (Lorenz and Smith, 2015; Schopp et al., 2017; Vitale et al., 2025). As new germplasm is introduced, regular updating of the reference population remains essential to preserve prediction reliability.

The phenotypic dataset represents an operational breeding program, characterized by unbalanced genotype representation, stage-specific replication, substantial environmental heterogeneity, and strong G × E interactions. While these features complicate genomic prediction, they are unavoidable in applied breeding. To address this complexity, a two-stage analytical framework was implemented. Environment-specific BLUEs obtained from spatially adjusted models, avoiding shrinkage and providing independent, high-quality inputs for genomic prediction. Excluding trials with very low heritability reduced noise and improved prediction reliability, though at the cost of environmental representation, highlighting the trade-off between data quality and connectivity. This approach aligns with best practices for large breeding programs using historical and unbalanced trial data (Piepho et al., 2008; Hussain et al., 2022; Khanna et al., 2022a, Khanna et al., 2022a, b).

### 4.2 Genomic prediction accuracies for grain yield and the role of G × E

GY prediction accuracies were 0.56 in five-fold cross-validation, consistent with previous GS studies in field pea and other pulses (Annicchiarico et al., 2017; Atanda et al., 2022; Castro-Urrea et al., 2023; Miller et al., 2023; Gebremedhin et al., 2024; Lin et al., 2025). However, under the more stringent LYO-CV scheme, which simulates forward prediction across breeding cycles, accuracies declined to 0.33–0.37, reflecting both the inherent complexity of GY as a polygenic trait and the substantial year-to-year environmental variation inherent to rainfed pulse production systems, rather than model inadequacy.

Most GY environment pairs (88%) exhibited low genetic correlations (r_g_ < 0.6), confirming a G × E interaction in the breeding program and highlighting that correlations and heritabilities varied considerably across years, underscoring the challenges posed by the quality and unbalanced nature of our historical data. Explicit modeling of G × E effects in the GBLUP framework improved GY prediction accuracy (up to +3.03%), consistent with findings in wheat (Burgueño et al., 2012; Jarquín et al., 2017) and barley (Cuevas et al., 2016; Lin et al., 2021). The relatively small gains observed likely reflect variable trial broad-sense heritability (0.2–0.97) and reduced environmental connectivity following exclusion of low-quality trials, and the stage-wise structure of breeding programs, where sparse early-generation testing constrains reliable estimation of environment-specific genetic effects (Rogers and Holland, 2022; Saludares et al., 2024). Under such conditions, large gains from G × E models are not expected; nevertheless, even incremental improvements from G × E models are valuable, as they provide insight into genotype adaptability and support more informed selection decisions.

Environmental clustering proved valuable for summarizing the large multi-environment data; however, differences in genomic prediction accuracy among clusters were driven primarily by genetic connectedness rather than by GY *per se*. Clusters with higher connectedness and greater genotype overlap across environments, notably Clusters 1 and 4, consistently achieved the highest prediction accuracy under both CV0 and CV1 scenarios, where CV1 is the true forward-prediction scenario. In contrast, clusters with fewer shared lines, such as Cluster 5, showed lower predictive performance despite distinct yield profiles. Such patterns reflect inherent constraints in breeding programs, including rapid germplasm turnover and unbalanced trial designs, and evolving breeding objectives. Despite these constraints, cluster-based predictions offer a practical framework for early-stage culling and prioritization, provided that inflated accuracy estimates arising from high connectedness are interpreted cautiously (Endelman et al., 2014; Isidro et al., 2015).

### 4.3 Leveraging correlated traits to improve grain yield prediction

Finally, exploiting genetic correlations among traits substantially improved GY prediction. Bivariate GBLUP models that correlate GY with biotic resistance traits (AB, BB, and BLRV; collected in the field) increased (~7.14%) accuracy from 0.56 (univariate) to as high as 0.60, consistent with previous pea studies (Annicchiarico et al., 2019; Bari et al., 2021; Zhao et al., 2022; Castro-Urrea et al., 2023). Gains in prediction accuracy were mainly driven by traits with moderately correlated traits (AB, BB), which reflect shared susceptibility pathways and the negative effect of disease severity on GY. Although PSbMV and BLRV have weak genetic correlations with GY, they are highly heritable and reliably phenotyped, allowing for accurate GEBV estimation. In bivariate models, these traits can indirectly reduce residual variance and stabilize GY predictions, though the improvement is limited by the historical breeding data structure. Secondary traits were phenotyped opportunistically, with uneven replication, strong environmental influences, and partial overlap with GY across environments. Operationally, univariate models remain the most reliable and scalable for forward prediction, especially in early generations when secondary trait data may be unavailable. These findings highlight the value of using correlated traits with high heritability and suggest incorporating secondary, high-throughput traits such as canopy temperature or NDVI to improve GY prediction in future breeding cycles (Jia and Jannink, 2012; Crain et al., 2018; Juliana et al., 2019; Zhao et al., 2022).

### 4.4 Genomic prediction accuracies for biotic stress resistance

Our study found that viral disease prediction accuracy ranged from moderate to high, with PSbMV and BLRV achieving the highest values (e.g., 0.42 in five-fold CV and up to 0.72 in LYO-CV). High predictive accuracy in PSbMV and BLRV, combined with monogenic inheritance resistance (Beck-Okins et al., 2022; Rubiales et al., 2023) enables early-stage selection based on GEBVs in large populations (e.g., segregating F2 populations), thereby reducing reliance on costly field or controlled-environment phenotyping.

In contrast, prediction accuracy for DM, BB, and AB was lower and more variable, reflecting their polygenic inheritance and strong environmental sensitivity. Phenotyping for these diseases is typically opportunistic, inconsistently replicated, and dependent on natural infection, and is further complicated by multiple pathogen species and diverse host-pathogen interactions. Single assessments cannot fully capture the disease spectrum, leading to reduced heritability and lower genomic prediction accuracy.

From a practical breeding perspective, improving genomic prediction accuracy requires extensive multi-environment phenotyping with relevant pathogen isolates to capture variability and support robust GS models. Updating models with new genotypic and phenotypic data, including isolate-specific details, is vital as pathogen populations evolve. Modern high-throughput phenotyping methods, such as UAV imaging, hyperspectral sensing, and measuring secondary traits related to disease, can generate large, standardized datasets across environments, increasing predictive accuracy and reducing reliance on labor-intensive traditional scoring (Ornella et al., 2012). By incorporating secondary and high-throughput traits, breeders can improve the predictive accuracy of polygenic disease resistance traits, facilitate more reliable GEBV-based selection, and accelerate the development of durable, disease-resistant cultivars.

### 4.5 Implementing genomic selection in field pea

**Figure 4:**
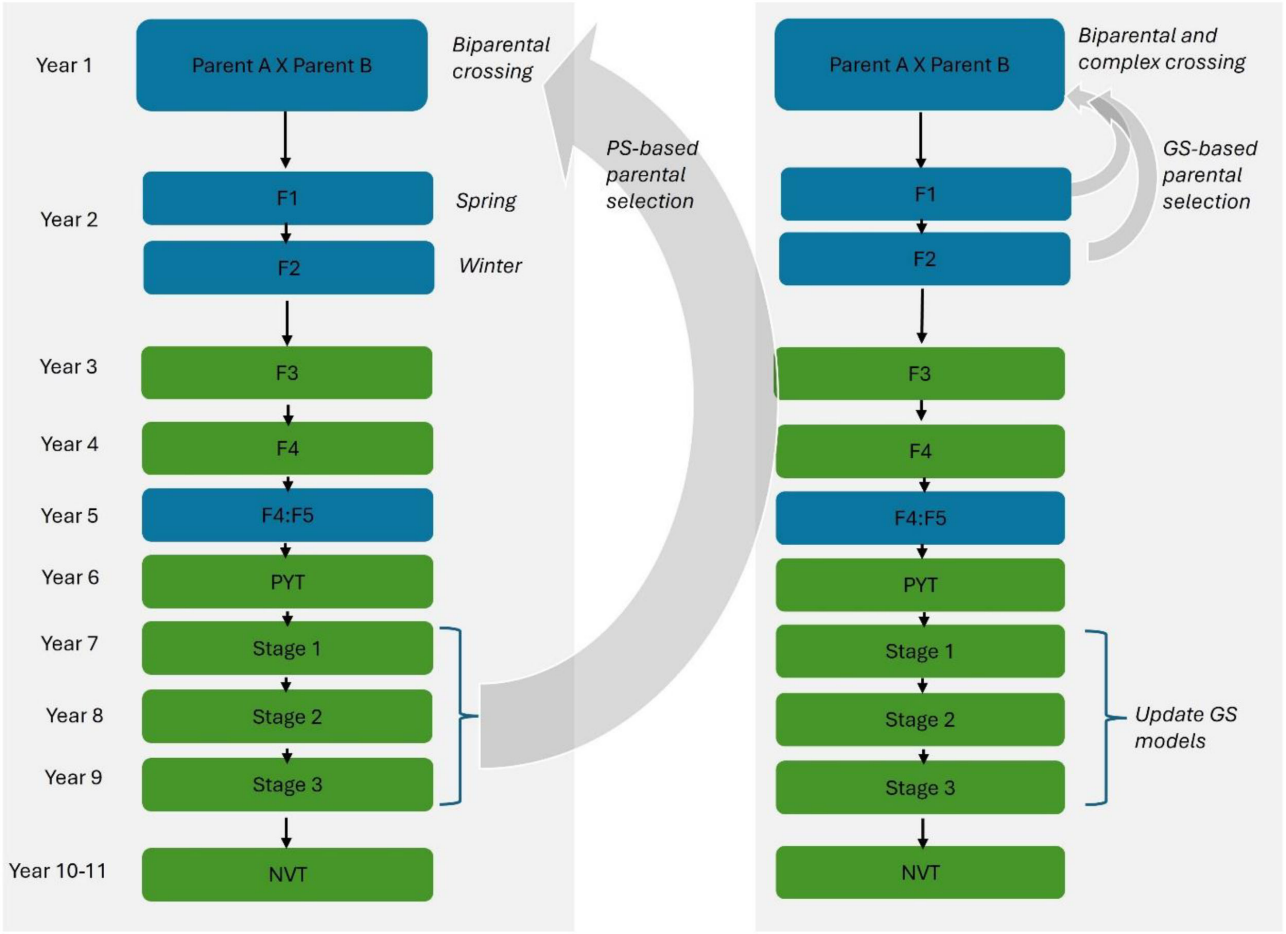
Schematic overview of the conventional (phenotypic) selection (PS; left) and genomic selection-based (GS; right) pipelines used in the Australian National Field Pea Breeding Program.

The results presented above demonstrate that genomic prediction can achieve useful accuracy for both grain yield and key disease resistance traits, providing a strong foundation for operational implementation in breeding. Historically, the National Field Pea Breeding Program followed a phenotypic selection pipeline comprising crossing, seed bulking, generation advancement, and multi-stage field testing for GY, disease resistance, abiotic stress tolerance, agronomic, and quality traits. Early-stage evaluation is conducted in single environments with partial replication, progressing to multi-environment trials with full replication, and ending with the National Variety Trials (NVT) for commercial release (Li et al., 2022). Parental selection typically occurs in advanced yield trials (Stage 2 and 3) based on GY, disease resistance, abiotic stress tolerance, and agronomic performance (**Figure 4**).

More recently, the breeding program has integrated GS, linear mixed models, and G × E modelling to shorten generation time, improve selection accuracy, and accelerate genetic gain. With GS, parents can be selected as early as the F_2_ generation, rather than at Stage 2 in the traditional pipeline. This substantially reduces the breeding cycle length (L), as defined by the Breeder’s Equation, while retaining the overall breeding framework. Implementing GS-based parent selection by year 2 reduces the time required to identify and recycle superior parents by more than three-fold compared with conventional phenotypic selection.

Prediction accuracy may decline when parents are from earlier generations due to shifting allele frequencies, decaying LD between markers and causal loci, and divergence between training and selection candidates (Jannink, 2010; Bastiaansen et al., 2012; Labroo and Rutkoski, 2022; Wientjes et al., 2022; Bandillo et al., 2023; Bernardo, 2025). This risk is further compounded because training populations are typically updated using phenotypic data from advanced yield trials (Stage 2 and 3), which may be genetically distant from early-generation material. To address these limitations, we introduced phenotyping in early generations (i.e., advanced yield trials - Stage 1), thereby improving genetic connectedness between training and selection populations. In addition, information from genetically correlated traits can also be incorporated via multivariate models, thereby increasing prediction accuracy in early-generation selection (Castro-Urrea et al., 2023). Sparse phenotyping strategies were also adopted to balance data quality, connectivity, and resource constraints. Regular incorporation of new phenotypic and genotypic data, along with recalibration of prediction models, remains essential to maintain predictive accuracy, as allele frequencies and LD change across selection cycles (Bernardo, 2025).

In the conventional pipeline, early-generation selections (e.g., F_2_ and F_4:5_; **Figure 4**) were primarily based on visual assessments, with approximately half of the lines proceeding to subsequent stages. This approach was constrained by strong environmental effects on phenotypes, limited trial capacity, and relatively low selection intensity. By contrast, GS now enabled the use of GEBVs at the F_4:5_ stage (~4,000–5,000 breeding lines; **Figure 4**), to efficiently cull poor performers. Consequently, only about 20–30% of lines advance to preliminary yield trials under GS, compared with ~50% under traditional phenotypic selection (Xu et al., 2020). This increase in selection intensity substantially reduces early-stage phenotyping costs, improves resource allocation, and increases the likelihood of retaining superior genetic combinations, ultimately translating into higher realized genetic gain (Atanda et al., 2021; Li et al., 2022; Gebremedhin et al., 2024; Saludares et al., 2024).

Furthermore, to enhance long-term genetic gain while maintaining diversity, the breeding program has implemented a computational approach to design cross-hybridization designs, enabling two cycles per year. We used a simulation tool, *CropSim* (developed in C++ by AVR in-house), to optimize parent selection and mating design and prevent rapid loss of genetic diversity due to inbreeding (Lin et al., 2017). *CropSim* takes the actual lines from the training population as inputs to simulate all possible cross combinations up to the F_8_ generation, thereby capturing genetic variance within families. Crosses are then ranked by simulated F_8_ GEBVs, and superior crosses are selected for future hybridization while maintaining diversity by limiting parent co-ancestry and fixed alleles (Li et al., 2022).

In traditional breeding cycles, inbreeding tends to accumulate because the field pea is an autogamous species, where repeated selfing rapidly fixes alleles, reduces heterozygosity, and narrows the effective population size (Johnson et al., 2024). This leads to a loss of genetic diversity, particularly when a small number of elite parents are repeatedly recycled. To mitigate these risks, alongside GEBVs, optimal haploid value (OHVs) (Daetwyler et al., 2015) are used, where GEBVs capture the expected average performance of a line’s progeny, and OHVs target the maximum long-term potential of parents by targeting favorable allele combinations that may not yet be fixed. Integrating both GEBV and OHV can prioritize parents that deliver short-term genetic, while also maintaining long-term diversity and adaptability. This integrated approach can be further enhanced by combining it with speed breeding (Watson et al., 2018) to further reduce the breeding cycle’s length without compromising genetic diversity.

Besides GY, breeders also simultaneously improve breeding germplasm for disease resistance, abiotic stress tolerance, quality, and a range of agronomic traits to achieve yield stability, often in the presence of unfavourable genetic correlations. Firstly, early-stage selection in large populations increases the probability of retaining favourable alleles for multiple traits (e.g., GY and protein content). Secondly, the implementation of the GS also enables formal multi-trait selection in early generations through selection indices, including weighted, tandem, index-based, and independent culling approaches. The effectiveness of these methods depends critically on trait weighting strategies and the stability of index performance under operational breeding conditions. Trait weights can be derived from economic values, desired genetic gains, or breeders’ defined priorities, each reflecting different risk–reward trade-offs and influencing correlated responses (Hazel, 1943; Batista et al., 2021; Covarrubias-Pazaran et al., 2022; Wellmann, 2023; Atanda and Bandillo, 2024). Poorly chosen weights or unaccounted G × E interactions can reduce gains in secondary traits or overall genetic improvement, emphasizing the need for cross-validation, sensitivity analyses, and periodic recalibration of indices.

GS-based selection indices have proven effective in optimizing antagonistic relationships, such as the trade-off between GY and protein content, and in combining complementary traits, such as GY and weed competitive ability in wheat (Michel et al., 2019b; Fradgley et al., 2023). Similar successes have been reported for GY, protein content, and rheological traits in wheat (Michel et al., 2018, 2019a), as well as for GY and oil content in safflower (Zhao et al., 2023). These approaches enable breeders to define and target specific product profiles that align with market demands at different stages of the breeding cycle. In the field pea program, the deployment of GS-based selection indices will facilitate efficient, simultaneous selection for yield, quality, stress tolerance, and disease resistance across thousands of candidates, accelerating the development of well-adapted cultivars.

Finally, our work improves understanding of the practical challenges in applying MET genomic prediction models to field pea breeding programs. This study shows that GS is shifting field pea breeding from traditional phenotypic selection to a more data-driven, predictive approach. By utilizing historical MET data, we demonstrate that GS can effectively target both simple and complex traits, especially when combined with G × E modeling, environmental clustering, optimal crossing, and multi-trait selection. Notably, GS significantly shortens breeding cycle times, enabling earlier, more informed decisions and greater resource efficiency. Although still in progress, integrating GS-based selection indices, optimal crossing strategies, and high-throughput phenotyping, including UAV-based assessments of agronomic and disease traits, will likely further improve genetic gain, preserve genetic diversity, and aid in developing resilient field pea varieties suited for future climate and production challenges.

## Data Availability

The original contributions presented in the study are included in the article/**Supplementary Material**. Further inquiries can be directed to the corresponding author.
