# Supplementary material for: Genomic prediction for grain yield and biotic stress resistance in field pea (Pisum sativum L.)
Source: Front Plant Sci. 2026 Apr 1;17:1739804. doi: 10.3389/fpls.2026.1739804 (PMC13079318; doi:10.3389/fpls.2026.1739804)
Supplement: Supplementary file 2 [file DataSheet2.pdf]

A

Year	Total genotypes per year	Unique genotypes per year
2013	407	106
2014	327	21
2016	406	321
2019	376	222
2020	369	134
2021	851	387
2022	845	473

B

Connectivity Matrix

	2019	2020	2016	2013	2014	2021	2022
2022	28	47	16	8	10	365	845
2021	50	139	17	11	11	851	365
2014	22	15	70	301	327	11	10
2013	19	12	65	407	301	11	8
2016	35	24	406	65	70	17	16
2020	138	369	24	12	15	139	47
2019	376	138	35	19	22	50	28

Supplementary Figure S1. Summary of genotype distribution and connectedness in multi-environment grain yield trials. (A) Table showing the total number of genotypes evaluated per year and the number of genotypes unique to each year. Unique genotypes, those not observed in any other year, are critical for assessing forward-prediction accuracy in CV1. (B) Connectivity matrix depicting the number of shared genotypes between years. Higher values indicate stronger genetic connectedness across years, which influences genomic prediction accuracy, particularly under CV0 scenarios.

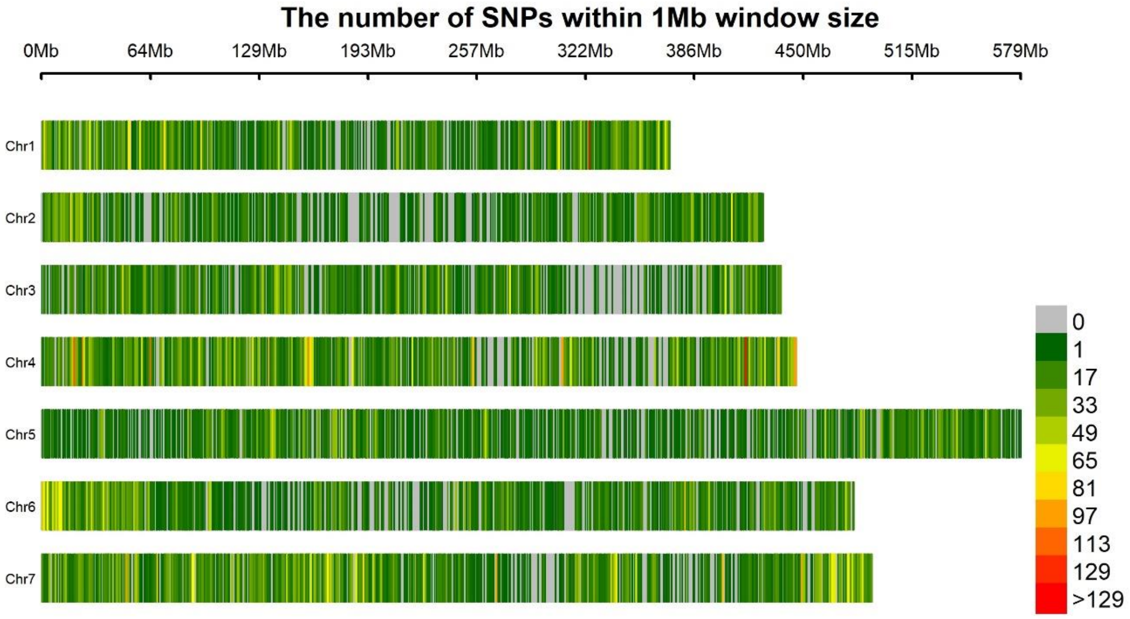

Supplementary Figure S2. Genome-wide distribution of 39,569 SNPs across seven field pea chromosomes using a 1 Mbp window. The number of SNPs per chromosome was as follows: Chr1 (5,263), Chr2 (3,976), Chr3 (4,488), Chr4 (7,858), Chr5 (4,900), Chr6 (5,446), and Chr7 (7,728).

A

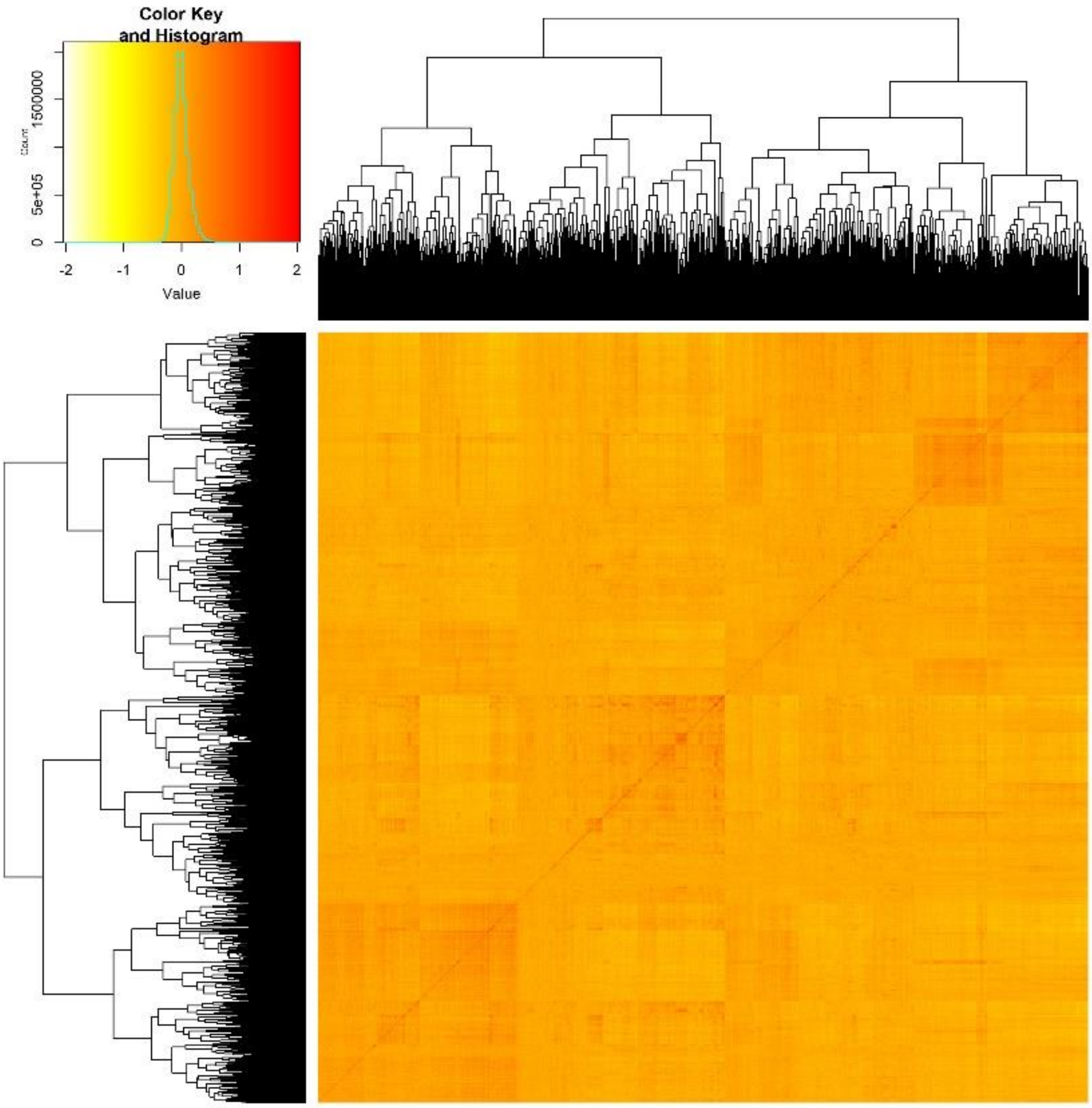

Color Key
and Histogram
Count
0
5e+05
1500000
-2
-1
0
1
2
Value

B

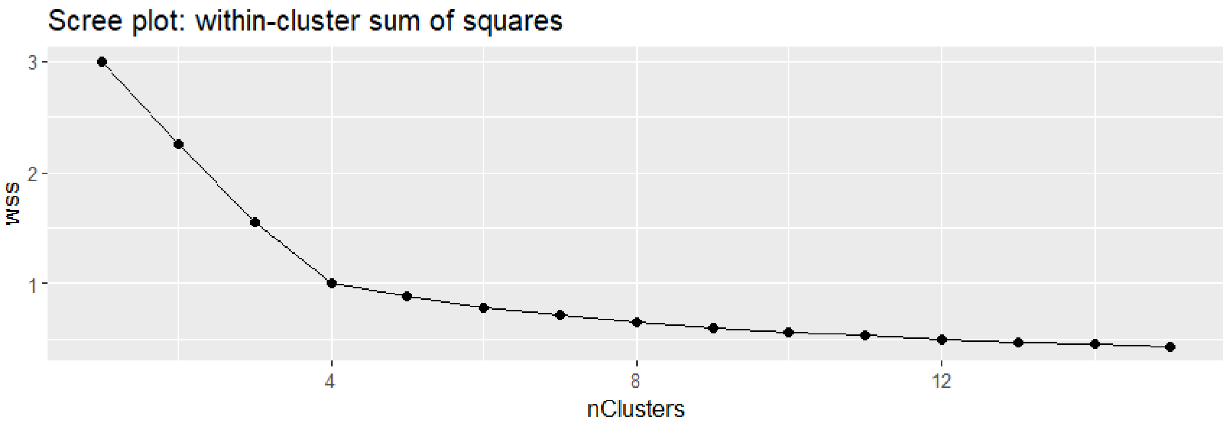

Scree plot: within-cluster sum of squares
wss
3
2
1
4
8
12
nClusters

C

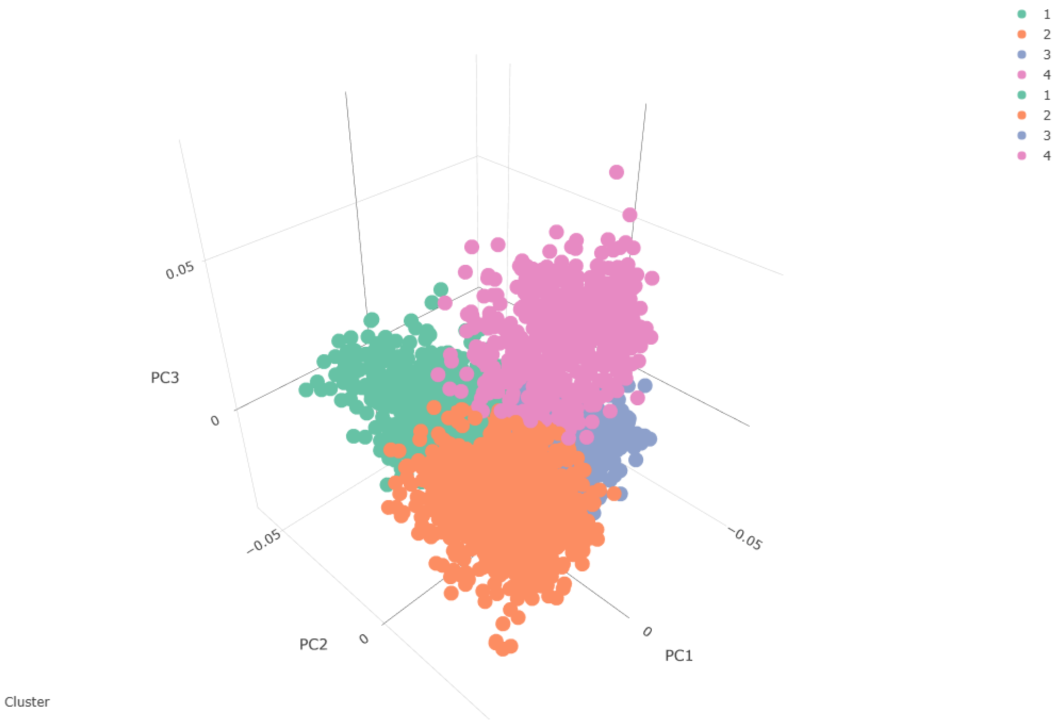

Supplementary Figure S3. Population structure and genomic relationships among field pea genotypes based on markers with a minor allele frequency (MAF) > 0.05. (A) Heat map of the genomic relationship matrix (G) computed using the VanRaden (2008) method, illustrating relatedness among 3,199 genotypes. Higher G values (red) indicate greater genetic similarity. (B) Scree plot showing the optimal number of clusters (n = 4) derived from principal component analysis (PCA). (C) Three-dimensional PCA plot visualized in the "plotly" package, revealing distinct clustering patterns among field pea genotypes, where the first three principal components explained 7.51%, 13.23%, and 17.27% of the total genomic variance, respectively.

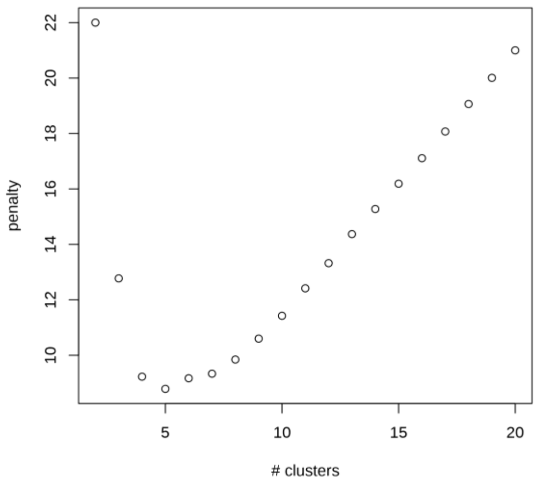

Supplementary Figure S4. Cluster penalty plot used to determine the optimal number of environment clusters. The Kelley–Gardner–Sutcliffe (KGS) penalty function was applied to the hierarchical clustering tree derived from Ward's method on the genetic correlation matrix of grain yield BLUEs across environments. The penalty score combines within-cluster dissimilarity (based on normalized deviations of individual and overall cluster means) with a penalty proportional to the number of clusters ($\alpha \times k$). The minimum penalty value indicates the optimal pruning level and the recommended number of clusters, providing an objective, reproducible criterion for selecting the number of clusters and avoiding subjective decisions.

Connectivity Matrix

	2	3	4	1	5
5	869	920	1257	1455	1776
1	661	1184	1072	1725	1455
4	742	843	1866	1072	1257
3	605	1184	843	1184	920
2	987	605	742	661	869

Supplementary Figure S5. The connectivity matrix between different clusters (1-5).

Supplementary Table S5. Descriptive summary of the grain yield across each cluster. ✓ represents presence of the data.

Year /State	Year /Site	Clusters				
		**1**	**2**	**3**	**4**	**5**
**Year**	2013	✓	✓	✓	✓	✓
	2014	✓	✓	✓	-	-
	2016	-	✓	-	✓	✓
	2019	✓	✓	✓	✓	✓
	2020	✓	-	✓	✓	-
	2021	✓	-	✓	-	✓
	2022	-	✓	-	✓	-
**State**						
Victoria	Sealake	✓	-	✓	-	✓
	Ouyen	-	✓	-	-	-
	Rupanyup	-	-	-	✓	-
	Beulah	✓	✓	-	✓	✓
	Curyo	✓	-	-	✓	✓
	Horsham	✓	-	-	✓	-
	Hopetoun	-	-	-	-	✓
South Australia	Kadina	✓	-	-	✓	✓
	Kingsford	✓	-	✓	✓	-
	Minnipa	-	✓	-	✓	✓
	Snowtown	✓	-	✓	✓	-
	Willamulka	-	✓	✓	-	-
Western Australia	Scadden	✓	✓	✓	-	-
	Salmon Gums	-	✓	-	-	-
New South Wales	Wagga Wagga	✓	-	✓	✓	-
	Yenda	✓	-	✓	✓	-
	Ardlethan	✓	-	✓	✓	-
	Balaklava	✓	✓	✓	✓	✓
